# Chromatin remodeler Znhit1 preserves hematopoietic stem cell quiescence by determining the accessibility of distal enhancers

**DOI:** 10.1038/s41375-020-0988-5

**Published:** 2020-07-21

**Authors:** Shenfei Sun, Ning Jiang, Yamei Jiang, Qiuping He, Hua He, Xin Wang, Li Yang, Runsheng Li, Feng Liu, Xinhua Lin, Bing Zhao

**Affiliations:** 1 State Key Laboratory of Genetic Engineering, School of Life Sciences, Zhongshan Hospital, Fudan University, Shanghai, 200438 China; 2 National Health Commission Key Laboratory of Reproduction Regulation, Shanghai Institute of Planned Parenthood Research, Shanghai, 200032 China; 3 State Key Laboratory of Membrane Biology, Institute of Zoology, Chinese Academy of Sciences, Beijing, 100101 China

**Keywords:** Cell signalling, Haematopoietic stem cells

## Abstract

Hematopoietic stem cell (HSC) utilizes its quiescence feature to combat exhaustion for lifetime blood cell supply. To date, how certain chromatin architecture and subsequent transcription profile permit HSC quiescence remains unclear. Here, we show an essential role of chromatin remodeler zinc finger HIT-type containing 1 (Znhit1) in maintaining HSC quiescence. We find that loss of Znhit1 leads to exhaustion of stem cell pool and impairment of hematopoietic function. Mechanically, Znhit1 determines the chromatin accessibility at distal enhancers of HSC quiescence genes, including *Pten*, *Fstl1*, and *Klf4*, for sustained transcription and consequent PI3K–Akt signaling inhibition. Moreover, Znhit1–Pten–PI3K–Akt axis also participates in controlling myeloid expansion and B-lymphoid specification. Our findings therefore identify a dominant role of Znhit1-mediated chromatin remodeling in preserving HSC function for hematopoietic homeostasis.

## Introduction

Transformations of chromatin architectures allowing transcriptional activation or repression of particular sets of genes are critical for stem cell fate determination [1, 2]. At the molecular level, this structural change can be brought by the replacement of canonical histone H2A with histone variant H2A.Z, which leads to chromatin remodeling and subsequent gene expression changes [3–8]. Our recent study has demonstrated that zinc finger HIT-type containing 1 (Znhit1), a component of SNF2-related CBP activator protein complex, incorporates H2A.Z for transcriptional regulation of stemness-related genes thus Lgr5+ stem cell maintenance [9]. To date, it is unknown whether Znhit1/H2A.Z functions in the fate determination of other tissue stem cells.

Hematopoietic stem cells (HSCs) lie at the apex of hematopoiesis hierarchy and continuously replenish lineage-committed progeny [10–12]. In adult bone marrow (BM), there are two major HSC populations: long-term HSCs (LT-HSCs) and short-term HSCs (ST-HSCs). LT-HSCs have the capacity of lifelong multilineage reconstitution, and most LT-HSCs stay in a dormant cell cycle or G0 phase that is termed as quiescence. ST-HSCs maintain the short-period (12 weeks) hematopoiesis by proliferating and differentiating into multipotent progenitors (MPPs) [13, 14]. Emerging evidence shows that quiescence preserves HSC function through preventing mutation, senescence, and exhaustion. Extrinsic and intrinsic factors such as cytokines, signaling pathways, and transcriptional factors (TFs) have been reported to restrict HSC in quiescent stage [15–18]. However, the mechanism of how certain chromatin architecture permits HSC quiescence remains unclear.

Pten, a dominant negative regulator of PI3K–Akt signaling, tightly restricts the cell cycle entry and progression of HSCs [19–21]. As a tumor suppressor gene, *Pten* is frequently mutated in malignancy, including leukemia that features dysregulated hematopoiesis. It has been reported that *Pten* transcription is determined by promoter-bound TFs (Egr1, PPAR-γ, and p53 for activation and Hes1 and Bmi1 for suppression) and promoter hypermethylation [22]. However, the roles of distal *cis*-regulatory element like enhancer or super-enhancer in sustaining *Pten* transcription thus HSC quiescence remain unknown. In particular, exploring how chromatin remodeling determines the accessibility of distal enhancer and consequence Pten expression should provide insights into the hematopoietic homeostasis and leukaemogenesis.

In this study, we employ Znhit1 conditional knockout mouse strain to investigate its roles in hematopoietic homeostasis. We show that Znhit1 restricts HSC in quiescent stage thus preserves its function. Znhit1 sustains the transcription of HSC quiescence genes (*Pten*, *Fstl1*, and *Klf4*) through modulating their distal enhancers. Notably, inhibition of activated PI3K–Akt signaling by LY294002 efficiently rescues the hematopoietic phenotype of Znhit1 deletion. Our findings establish the central role of Znhit1 in regulating HSC quiescence, which well explains how chromatin remodeler ensures proper signaling at transcriptional level for HSC fate determination and hematopoietic homeostasis.

## Materials and methods

### Mice

*Znhit1*^fl/fl^ mice were generated by Model Animal Research Center of Nanjing University (Nanjing, China) [9]. *Mx1-cre* mice were obtained from the Jackson Laboratory. *H2afv*^fl/fl^/*H2afz*^fl/fl^ mice were obtained from RIKEN BioResource Center. All strains were maintained in C57BL/6 background.

For Cre induction, 6–8 weeks old mice were intraperitoneally injected with 300 μg polyinosinic–polycytidylic acid (pIpC) (Novus) every 2 days for three times. For LY294002 administration, mice were daily injected with 2 mg of LY294002 (Selleck) for 7 days after pIpC treatment.

All breeding and experimental procedures were performed in accordance with the relevant guidelines and regulations and with the approval of the Animal Care and Use Committee at Fudan University.

### Flow cytometry

Single-cell suspensions prepared from BM, peripheral blood (PB), and spleen were incubated for 30 min at 4 °C with fluorochrome–antibodies (BioLegend). Dead cells were excluded by FVD eFluor® 455UV (eBioscience) staining. Flow cytometry was performed using CytoFLEX (Beckman), LSR Fortessa, or FACSAria II (BD) flow cytometer. Antibodies used in this study are available in Supplementary Methods.

For cell cycle or quiescence analysis of HSCs, the BM cells were incubated with fluorochrome–antibodies, fixed and permeabilized with Transcription Factor Buffer Set (BD), and stained with 5 μg/ml DAPI (Sigma) or PE-anti-Ki67 (BioLegend).

For Akt phosphorylation assay, live Lin^−^ BM cells were sorted, starved in 2% FBS/IMDM (Gibco) for 1 h, and then treated with 5 ng/ml GM-CSF (Peprotech) at 37 °C for indicated time. After stimulation, the cells were fixed, permeabilized, and stained with APC-Cy7-anti-CD117, PerCP-Cy5.5-anti-Sca1, anti-p-Akt (Cell Signaling), and PE-anti-IgG (eBioscience).

### RNA-seq and ATAC-seq

Freshly sorted Lin^−^Sca1^+^cKit^+^ cells (LSKs) (1.0 × 10^4^) were subjected to RNA-seq or ATAC-seq. Additional details are provided in Supplementary Methods.

### Statistical analysis

We employed Student’s *t* test or ANOVA test to analyze the parametric experimental results. In nonparametric data analysis, we employed Wilcoxon’s rank sum test for two-group and Kruskal–Wallis’ H test for multigroup. Significant differences were noted with asterisks.

## Results

### Znhit1 deletion leads to acute expansion of HSCs

**Fig. 1:**
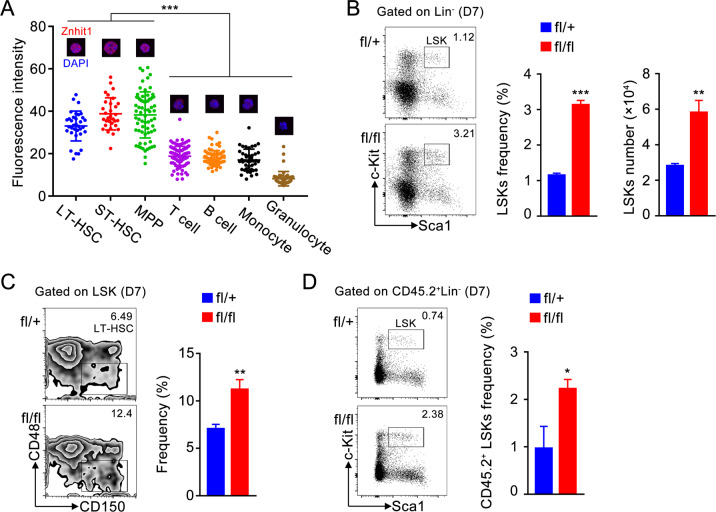
Znhit1 deletion leads to acute expansion of HSCs. **a** Hematopoietic lineages were sorted for examining the protein level of Znhit1. Fluorescence intensity of cells was calculated and shown on the point. **b** Flow cytometry of BM cells showed the gate, frequency, and number of LSKs from control (fl/+) and *Znhit1*^−/−^ (fl/fl) mice at D7 (day 7) following pIpC treatment (*n* (the number of mice in each group) = 3). **c** Flow cytometry of BM cells showed the gate and frequency of SLAM LT-HSC from control (fl/+) and *Znhit1*^−/−^ (fl/fl) mice at D7 following pIpC treatment (*n* = 3). **d** Flow cytometry of BM cells showed the gate and frequency of CD45.2^+^ LSKs from chimeric mice at D7 following Znhit1 deletion (*n* = 3). Results were representative of at least three independent experiments. Data were presented as mean ± s.d. *p < 0.05; **p < 0.01; ***p < 0.001.

To determine the expression pattern of Znhit1 in adult hematopoietic system, we sorted different lineages from 6-week-old C57BL/6 mouse BM or PB and examined Znhit1 mRNA and protein level. As shown in Fig. 1a and Supplementary Fig. 1a–c, HSCs and progenitors had enriched Znhit1 expression, while differentiated cells (granulocytes, monocytes, T cells, and B cells) had significantly reduced Znhit1 expression, suggesting Znhit1 might be involved in the regulation of hematopoiesis.

To investigate the functions of Znhit1 in maintaining hematopoietic homeostasis, we generated *Znhit1*^fl/fl^; *Mx1-cre* mice to achieve an inducible hematopoietic knockout of Znhit1 upon pIpC treatment. Examination of Znhit1 mRNA and protein levels confirmed the Znhit1 deletion in HSC-enriched LSK cells (Supplementary Fig. 1d, e). Flow cytometric analysis revealed that Znhit1 deficiency led to dramatic expansion of LSKs in BM (from 1.12 to 3.21%) 7 days following pIpC injection (Fig. 1b). Interestingly, we observed an elevated frequency of LT-HSCs (from 10.17 ± 0.29 to 18.83 ± 1.214% in LSKs) but not ST-HSCs or MPPs (Supplementary Fig. 2a), suggesting the LT-HSCs were most sensitive to Znhit1 deficiency. SLAM-receptor-based phenotypic analysis (CD48^−^CD150^+^LSKs) revealed that both the frequency and the number of LT-HSCs were significantly increased after Znhit1 deletion (Fig. 1c and Supplementary Fig. 2b). These results indicate that Znhit1 tightly restricts the population of LSKs, especially LT-HSCs, in adult hematopoietic system.

To verify that Znhit1 functions in a lineage-autonomous manner, we transplanted 1.0 × 10^6^ CD45.2^+^ *Znhit1*^fl/fl^; *Mx1-cre* donor cells into lethally irradiated CD45.1^+^ recipients. After 6 weeks, pIpC was injected into chimeric mice to induce Znhit1 deletion. Consistently, mice receiving Znhit1-deficient donor cells displayed a significant increase in the frequency of LSKs 7 days following pIpC treatment (Fig. 1d).

### Znhit1 is essential for HSC quiescence maintenance

Given that the expansion of HSCs could be due to either enhanced proliferation or blocked differentiation, we further examined how Znhit1 determines the fate of HSCs.

Znhit1-deficient mice did not show obvious defect in the differentiation of myeloid progenitor (common myeloid progenitors (CMPs), granulocyte/macrophage progenitors (GMPs), and megakaryocyte/erythrocyte progenitors (MEPs)) or lymphoid progenitor (lymphoid-primed MPPs (LMPPs) and common lymphoid progenitors (CLPs)) (Supplementary Fig. 2d–f).

**Fig. 2:**
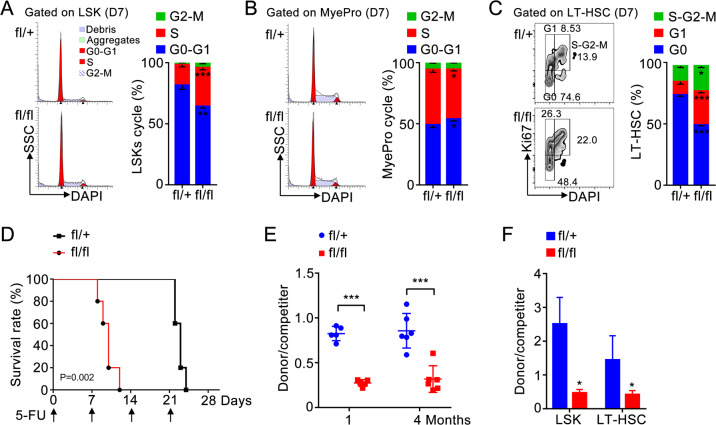
Znhit1-mediated quiescence preserves HSC functions. Flow cytometry of BM cells showed the gates and frequencies of the cell cycle in LSKs (**a**) or myeloid progenitors (MyePro) (**b**), stained with DAPI, from control (fl/+) and *Znhit1*^−/−^ (fl/fl) mice at D7 following pIpC treatment (*n* = 3). **c** Flow cytometry of BM cells showed the gates and frequencies of proliferation and G0 phase in LT-HSC, stained with Ki67, from control (fl/+) and *Znhit1*^−/−^ (fl/fl) mice at D7 following pIpC treatment (*n* = 3). **d** Kaplan–Meier survival curves of control (fl/+) and *Znhit1*^−/−^ (fl/fl) mice after weekly injections (shown by arrows) of 5-FU following pIpC treatment (*n* = 5). **e** Analysis of the donor (CD45.2^+^) contribution to PB 1 month and 4 months following competitive BM transplantation. **f** Analysis of the donor (CD45.2^+^) contribution to LSK and LT-HSC 4 months following competitive BM transplantation (*n* = 5). Results were representative of at least three independent experiments. Data were presented as mean ± s.d. *p < 0.05; **p < 0.01; ***p < 0.001.

To testify whether Znhit1 deletion promotes HSCs expansion through facilitating its proliferation, we assessed the role of Znhit1 in controlling the cell cycle of LSKs. DAPI staining revealed that 81.5% LSKs stayed in G0/G1 phase under homeostatic condition, while only 64.2% remained after Znhit1 deletion (Fig. 2a). This indicates that Znhit1 prevents LSKs from fast cycling. Moreover, Znhit1 deficiency had an opposing effect on proliferation of myeloid progenitors (Fig. 2b), suggesting the antiproliferative effect of Znhit1 is LSKs specific.

Distinct from cycling ST-HSCs or MPPs, most LT-HSCs prefer to stay in quiescent G0 phase [13]. Since expansion of LT-HSCs responded to Znhit1 deficiency most sensitively, we set out to examine whether Znhit1 deletion disrupted the quiescence maintenance of LT-HSCs. We sorted LT-HSCs and employed Ki67 (specifically expresses in proliferating but not G_0_ cells [13]) to distinguish quiescence. As shown in Fig. 2c, 74.6% control LT-HSCs while only 48.4% *Znhit1*^−/−^ LT-HSCs were quiescent, demonstrating that Znhit1 is essential for maintaining the quiescence of LT-HSCs.

### Znhit1-mediated quiescence preserves HSC functions

Quiescence is a functional characteristic of multiple tissue stem cells to prevent mutation, senescence, and exhaustion [17]. Previous studies have shown that quiescent HSCs have superior long-term engraftment potential relative to that of actively cycling HSCs [23]. Indeed, Znhit1-deficient LSKs tended to undergo apoptosis, which was revealed by increased annexin V staining (Supplementary Fig. 2c). To further address whether Znhit1-mediated quiescence preserves HSC populations, we followed the behaviors of LT-HSCs, ST-HSCs, and MPPs to 30 days post Znhit1 deletion. Immunophenotypic quantification showed that Znhit1-deficient mice had significantly fewer ST-HSCs (0.05% (*Znhit1*^−/−^) versus 0.10% (*Znhit1*^fl/+^)) and MPPs (0.03% (*Znhit1*^−/−^) versus 0.11% (*Znhit1*^fl/+^)) in BM 30 days following pIpC injection (Supplementary Fig. 2g), indicating that Znhit1 indeed prevents ST-HSCs and MPPs from exhaustion.

As LT-HSCs exhaustion was not observed by phenotypic analysis (Supplementary Fig. 2g), we characterized the long-term hematopoietic ability of LT-HSCs through ablating cycling HSCs with chemotherapeutic agent 5-fluorouracil (5-FU). Weekly challenged with 5-FU, Znhit1-deficient mice showed dramatically decreased survival compared with littermate controls (Fig. 2d), demonstrating that Znhit1-mediated quiescence preserves the long-term hematopoietic function of LT-HSCs.

We further examined the multilineage reconstitution ability of Znhit1-deficient HSCs by performing competitive transplantation experiments. CD45.2^+^ control or *Znhit1*^−/−^ BM cells were transplanted into lethally irradiated recipient mice together with CD45.1^+^ competitors at 1:1 ratio. Although 7-day Znhit1 deletion tripled the number of LSKs at the time of transplantation, *Znhit1*^−/−^ BM cells showed significantly decreased PB reconstitution potential since 1 month post transplantation (Fig. 2e). Znhit1 deficiency led to impaired reconstitution of multiple lineages, including B cells, T cells, monocytes, and granulocytes (Supplementary Fig. 2h). In addition, fewer LSKs even LT-HSCs could be detected in the absence of Znhit1 4 months post transplantation (Fig. 2f), which is in consistent with the idea that Znhit1 prevents stem cell pool from exhaustion. These data together demonstrate that Znhit1-mediated quiescence secures the long-term self-maintenance and multilineage reconstitution abilities of HSCs.

### Znhit1 sustains the transcription of HSC quiescence genes to restrict PI3K–Akt signaling for HSC fate determination

**Fig. 3:**
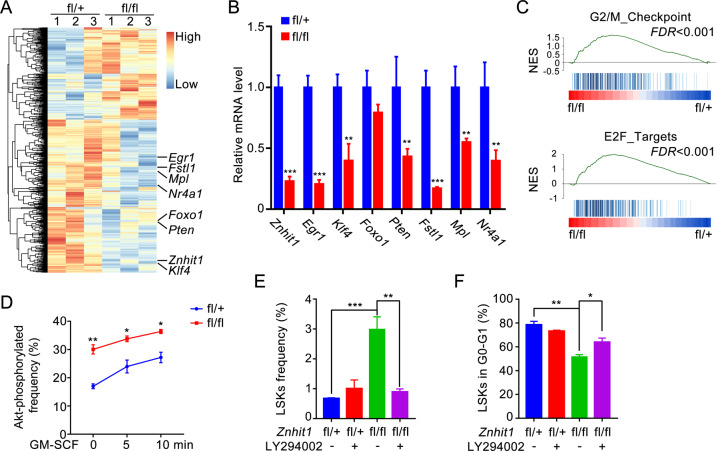
Znhit1 sustains the transcription of HSC quiescence genes to restrict PI3K–Akt signaling for HSC fate determination. **a** Clustered heatmap of differentially expressed genes after Znhit1 deletion. Indicated genes were marked in right. **b** qRT-PCR was performed to show indicated genes expression in LSKs from control (fl/+) and *Znhit1*^−/−^ (fl/fl) mice at D5 following pIpC treatment. For qRT-PCR, H3 was used as an internal control (*n* = 3). **c** Gene set enrichment analysis (GSEA) of selected gene sets encoding products related to G2/M checkpoint, or E2F targets, presented as normalized enrichment score (NES). Gene expression data come from control (fl/+) and *Znhit1*^−/−^ (fl/fl) LSKs. **d** Flow cytometry of the frequency of Akt-phosphorylated (Ser473) in control (fl/+) and *Znhit1*^−/−^ (fl/fl) LSKs untreated or stimulated with GM-CSF for 5 or 10 min (*n* = 3). **e** At D7 following Znhit1 deletion, flow cytometry of BM cells was performed to show the frequency of control (fl/+) and *Znhit1*^−/−^ (fl/fl) LSKs with/without daily LY294002 treatment for total 7 days after pIpC injection (*n* = 3). **f** At D7 following Znhit1 deletion, flow cytometry of BM cells was performed to show the frequency of control (fl/+) and *Znhit1*^−/−^ (fl/fl) LSKs in G0-G1 phase with/without daily LY294002 treatment for total 7 days after pIpC injection (*n* = 3). Results were representative of at least three independent experiments. Data were presented as mean ± s.d. *p < 0.05; **p < 0.01; ***p < 0.001.

To understand the underlying mechanisms of how Znhit1 maintains HSCs quiescence, LSKs were sorted from 2-month-old control and *Znhit1*^−/−^ mice at day 5 after pIpC administration then subjected to gene transcriptome examination (Supplementary Fig. 3a, b). RNA-sequencing data from three biological replicates revealed a set of 1035 differentially expressed genes in Znhit1-deficient LSKs (Fig. 3a, *p* < 0.05 and fold change ≥ 1.8). Gene Ontology analysis highlighted intensive expression alteration of genes involved in the regulation of cell cycle (Supplementary Fig. 3c). Especially, Znhit1 deletion ablated the expression of several critical genes maintaining LT-HSCs quiescence, including *Egr1*, *Klf4*, *Pten, Fstl1, Mpl*, and *Nr4a1* [19, 20, 24–28] (Fig. 3a and qPCR verification in Fig. 3b). Consistently, gene set enrichment analysis indicated that Znhit1-deficient LSKs had decreased enrichment of LT-HSC signatures but increased enrichment of G2/M checkpoint and E2F targets (Fig. 3c and Supplementary Fig. 3d). These results reinforce our conclusion that Znhit1 deletion disrupts HSC quiescence.

Among the Znhit1-sustained HSC quiescence genes, *Pten* and its inducer *Egr1* were reported to govern cell cycle through suppressing PI3K–Akt signaling [19, 20]. Therefore, we hypothesized that Znhit1 deletion might disrupt HSC quiescence via PI3K–Akt pathway. To test this, we first evaluated the PI3K–Akt signaling activity, represented by Akt phosphorylation level, in control and *Znhit1*^−/−^ LSKs. Flow cytometric phosphoprotein analysis showed that the Akt phosphorylation level of untreated *Znhit1*^−/−^ LSKs was even higher than that of GM-CSF-treated control LSKs (Fig. 3d and Supplementary Fig. 3e), indicating that Znhit1 deficiency is sufficient to induce Akt hyperactivation.

To further support the notion that Znhit1 deficiency disrupts HSCs quiescence through activating PI3K–Akt signaling, we examined whether the PI3K-specific inhibitor LY294002, which blocks Akt activation [29], could rescue the phenotypes of Znhit1 deletion. The data in Fig. 3e demonstrated that daily injection of LY294002 efficiently abolished the promoting effect of Znhit1 deficiency on acute HSC expansion. In accordance, Znhit1-deficient LSKs were protected from aberrant proliferation in the presence of LY294002 (Fig. 3f). Taken together, these results suggest that Znhit1 sustains the transcription of HSC quiescence genes to restrict PI3K–Akt signaling for HSC fate determination.

### Znhit1 modulates the chromatin accessibility of distal enhancers for transcriptional regulation

**Fig. 4:**
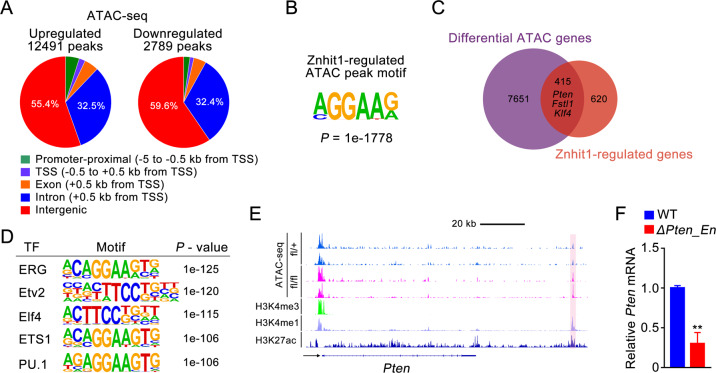
Znhit1 modulates the chromatin accessibility of distal enhancers for transcriptional regulation. **a** Distribution of differentially accessible ATAC peaks on genome in control and *Znhit1*^−/−^ LSKs at D5 following pIpC treatment. **b** Sequence motif identified within Znhit1-regualted ATAC peaks. **c** Venn diagram showing the overlap between differential peak genes from ATAC-seq and differentially expressed genes from RNA-seq following Znhit1 knockout. **d** Motif analysis of differential ATAC peaks in Znhit1-regulated genes for putative transcription factor (TF)-binding sites by using HOMER database. **e** ATAC-seq signals and ChIP-seq signals for H3K4me3, H3K4me1, and H3K27ac binding at *Pten*, and ChIP-seq data come from published data [34]. **f** Deletion of distal enhancer downregulated *Pten* expression in EML cells. Results were representative of at least three independent experiments. Data were presented as mean ± s.d. **p < 0.01.

Gene transcription requires chromatin accessibility at locus [30]. To determine whether Znhit1-mediated chromatin remodeling could alter the accessibility at HSC quiescence gene loci, we performed the assay for transposase-accessible chromatin with sequencing (ATAC-seq) in LSKs sorted from control and *Znhit1*^−/−^ mice. By analyzing the ATAC-seq data from two biological replicates, we identified 51,037 accessible chromatin regions (indicated by ATAC peaks) in control and 71,336 accessible chromatin regions in Znhit1-deficient LSKs (Supplementary Fig. 4a). Comparing with previous HSC document [31], we observed correlated ATAC peaks and comparable signal-to-noise ratio (represented by *Ifng* locus in Supplementary Fig. 4b), which validated the data quality. Further analysis revealed that Znhit1 deletion led to 15,280 changes in chromatin accessibility (12,491 sites opened and 2789 sites closed), which are significantly enriched in MGGAAR motif (M = A/C; R = G/A) (Fig. 4a, b). Interestingly, these altered ATAC peaks accumulated in introns and intergenic regions (Fig. 4a), indicating that Znhit1 mainly modulates the accessibility of regulatory chromatin regions in HSCs.

The altered ATAC peaks were annotated to a total of 8066 genes. Comparing them with 1035 Znhit1-regulated genes identified by RNA-seq, we found that 415 Znhit1-regulated genes, including *Pten*, *Fstl1*, and *Klf4*, had substantial changes in chromatin accessibility (Fig. 4c and gene list in Supplementary Table 1). Motif enrichment assay revealed that the Znhit1-remodeled chromatin regions with regulatory effect showed specific binding to a set of TFs, including ERG, Etv2, Elf4, ETS1, and PU.1 (Fig. 4d). Of note, activation of the top-ranking factor PU.1 has been reported to accelerate cell division of HSCs [32]. These results suggested that Znhit1-mediated chromatin remodeling might determine the access of hematopoietic TFs for transcriptional regulation.

TFs bind *cis*-regulatory elements to exert transcriptional regulation functions [33], which promoted us to address the identity of Znhit1-remodeled chromatin regions at HSC quiescence gene loci. Znhit1 deletion led to increased accessibility of indicated distal regions of *Pten*, *Fstl1*, and *Klf4*. Remarkably, these opened chromatin regions were specifically enriched with H3K4me1 and H3K27ac landmarks but not H3K4me3 landmark [34] (Fig. 4e and Supplementary Fig. 4d, e), supporting that the Znhit1-remodeled distal regions are active enhancers. We then took *Pten* as an example to evaluate the function of identified active enhancers in boosting gene transcription. As expected, removal of distal enhancer by CRISPR/Cas9 ablated the transcription of *Pten* in erythroid myeloid lymphoid (EML) cells and NIH3T3 cells (Fig. 4f and Supplementary Fig. 4f–h). Taken together, we demonstrate that Znhit1 sustains the transcription of HSC quiescence genes through modulating the chromatin accessibility of distal enhancers.

### Znhit1 downstream effector H2A.Z sustains *Pten* transcription

**Fig. 5:**
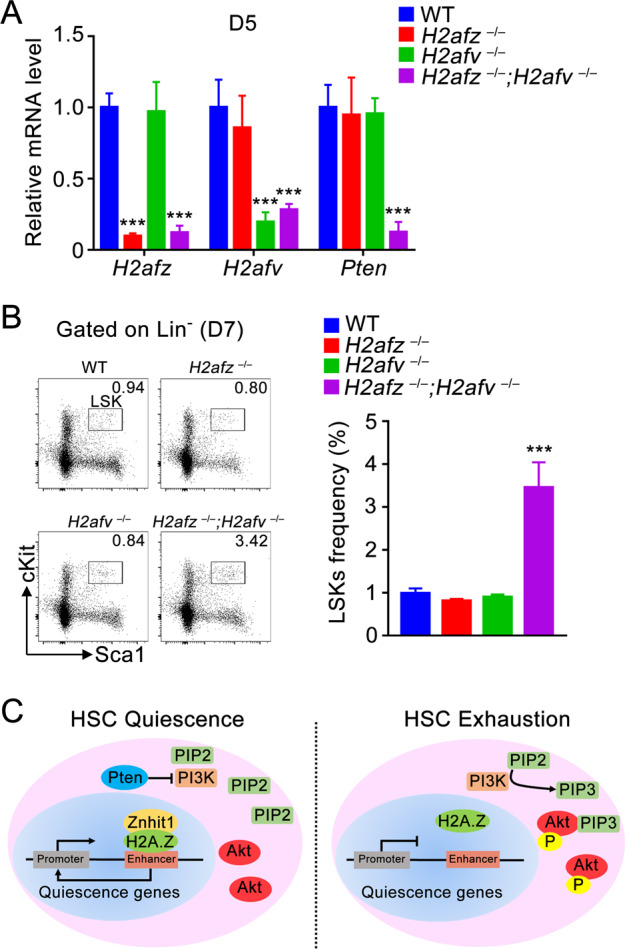
Znhit1 downstream effector H2A.Z sustains Pten transcription. **a** qRT-PCR was performed to show *H2afz*, *H2afv*, and *Pten* expression in WT, *H2afz*^−/−^, *H2afv*^−/−^, and *H2afz*^−/−^; *H2afv*^−/−^ LSKs at D5 following pIpC treatment. **b** Flow cytometry of BM cells showed the gate and frequency of LSKs from WT, *H2afz*^−/−^, *H2afv*^−/−^, and *H2afz*^−/−^; *H2afv*^−/−^ mice at D7 following pIpC treatment (*n* = 3). **c** Working model of how Znhit1 regulates quiescence genes for HSC fate determination. Results were representative of at least three independent experiments. Data were presented as mean ± s.d. ***p < 0.001.

Our recent in vivo study showed that Znhit1 controls gene transcription through depositing histone variant H2A.Z for chromatin remodeling [9]. Indeed, as represented by *Pten* locus, the Znhit1-regulated accessible chromatin regions were specifically occupied by enriched H2A.Z (Supplementary Fig. 4c) [35]. We employed H2A.Z hematopoietic knockout mice to further assess the significance of Znhit1 downstream effector H2A.Z in *Pten* transcription regulation. H2A.Z has two isoforms in mouse, H2afv and H2afz, which are encoded by separated loci [36]. Knockout of both isoforms, but not either single, efficiently suppressed the expression of *Pten* (Fig. 5a), indicating the redundant role of H2afv and H2afz in controlling gene transcription. Consistently, *H2afv* and *H2afz* double deletion, but not either single, led to LSKs acute expansion (Fig. 5b), which well mimicked the phenotype of Znhit1 deficiency. These data demonstrated the essential role of H2A.Z in sustaining *Pten* transcription for HSC quiescence, supporting that Znhit1 and downstream effector H2A.Z cooperate to achieve tissue-specific transcriptional regulation.

### Znhit1–Pten–PI3K–Akt axis controls myeloid expansion and B-lymphoid specification

**Fig. 6:**
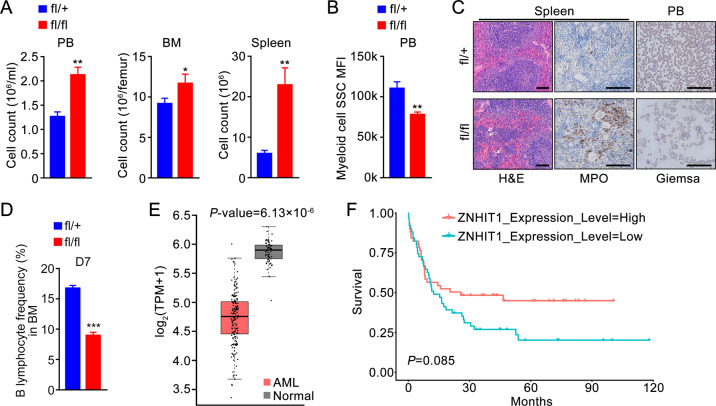
Znhit1 controls myeloid expansion and B-lymphoid specification. **a** Absolute myeloid cell numbers of PB, BM, and spleen from control (fl/+) and *Znhit1*^−/−^ (fl/fl) mice. **b** The SSC MFI of PB myeloid cell from control (fl/+) and *Znhit1*^−/−^ (fl/fl) mice. **c** Hematoxylin-eosin (H&E)-stained spleen sections, MPO-stained spleen sections, and Giemsa-stained blood cells from control (fl/+) and *Znhit1*^−/−^ (fl/fl) mice. Arrows showed myeloid cells. Scale bar, 50 μm. **d** Frequency of B lymphocyte from control (fl/+) and *Znhit1*^−/−^ (fl/fl) mice. **e** Expression level of ZNHIT1 in the human AML data set. **f** Overall survival in AML patients stratified by ZNHIT1 expression. Results were representative of at least three independent experiments. Data were presented as mean ± s.d. *p < 0.05; **p < 0.01; ***p < 0.001.

In addition to disturbed HSCs quiescence, Pten-deficient mice show myeloid and T-lymphoid expansion and develop myeloproliferative disorder (MPD) [19, 20]. As Znhit1 maintains HSC quiescence through controlling Pten expression, we further determined whether Znhit1 deletion led to myeloid expansion and malignant hematopoiesis. We observed dramatically increased myeloid cells (CD11b^+^Gr1^+^) in BM, PB, and spleen of *Znhit1*^fl/fl^; *Mx1-cre* mice 30 days following pIpC administration (Fig. 6a and Supplementary Fig. 5a). Of note, the SSC median fluorescence intensity (MFI) of myeloid cells in PB was significantly reduced, which indicated that Znhit1 deletion abolished the maturation of myeloid lineage (Fig. 6b and Supplementary Fig. 5b). Besides, hematopoietic Znhit1 deficiency resulted in splenomegaly and disorganized splenic structure, which associated with myeloid cells infiltration and myeloperoxidase (MPO)-positive cells increase (Fig. 6c and Supplementary Fig. 5c). Flow cytometric analysis and colony-forming units culture assay revealed the presence of HSCs and myeloid-forming splenocytes in spleen of Znhit1-deficient mice, indicating that hematopoietic Znhit1 deletion led to extramedullary hematopoiesis (Supplementary Fig. 5d, e). These data together supported that Znhit1-deficient mice developed MPD-like disorder [37]. Moreover, blocking progressive AKT activation by LY294002 significantly rescued the increased myeloid cell numbers in *Znhit1*^−/−^ BM cells (Supplementary Fig. 5h), which suggested that Znhit1 deletion might induce MPD through silencing Pten.

We also noticed a significant decrease of B cells in PB of Znhit1-deficient mice (Supplementary Fig. 5f). Further flow cytometric analysis revealed that 7-day deletion of Znhit1 was sufficient to block B-lymphoid specification (Fig. 6d and Supplementary Fig. 5g). This effect was independent of HSCs lymphoid differentiation as the frequency of LMPPs or CLPs was not affected 7 days after Znhit1 deletion (Supplementary Fig. 2e, f). To examine whether Znhit1/H2A.Z controls B-lymphoid specification via Pten–PI3K–Akt pathway, we administrated LY294002 and found that PI3K–Akt inactivation efficiently recused B-lymphoid contraction caused by Znhit1 deficiency (Supplementary Fig. 5i). In summary, we established the critical roles of Znhit1–Pten–PI3K–Akt axis in controlling myeloid expansion and B-lymphoid specification.

To determine whether ZNHIT1 participates in human acute myeloid leukemia (AML) progression, we analyzed the mRNA sequencing data of 173 adult AML samples (Ley et al. [38]) and 70 normal blood cell samples (Genotype-Tissue Expression Project). We found that ZNHIT1 expression is significantly downregulated in AML compared with normal (Fig. 6e). Furthermore, ZNHIT1 expression positively correlated with the survival of AML patients (Fig. 6f). These results suggest that loss of ZNHIT1 expression might be a risk factor of AML.

## Discussion

In adults, homeostasis between the quiescent and cycling states of HSCs is essential to balance self-maintenance and lineage reconstitution. Here, we examine the roles of Znhit1 in hematopoietic homeostasis and demonstrate that Znhit1 is essential for HSC fate determination. Our experimental data favor a model for Znhit1 in maintaining HSC quiescence: Znhit1 incorporates H2A.Z to modulate distal enhancers for transcriptional regulation of HSC quiescence genes; Znhit1 deficiency leads to PI3K–Akt signaling activation, which drives the quiescence to proliferation transition of HSCs (Fig. 5c).

HSC quiescence is fine-tuned by cell cycle regulators and signaling pathways [15, 18]. Multiple transcription factors, including Foxo1/3/4 [39], PU.1 [40], and Satb1 [41], play critical roles in maintaining HSC quiescence through programming specific transcriptions. To access transcription factors, chromatin needs to be modulated by histone modifications and architecture remodeling. Recent reports showed that chromatin remodeler Arid1a and Bptf regulate the expression of HSC stemness genes [42, 43]. However, Ikzf2, which builds the chromatin accessibility essential for leukemia stem cells, is dispensable for HSC maintenance [44]. These studies suggest that the functions of chromatin remodeling in HSC fate determination is context dependent. We now demonstrate that Znhit1 preserves HSC quiescence by remodeling the distal chromatin for transcriptional regulation, which elucidates how chromatin remodeling factor ensures proper signaling activity at transcriptional level for HSC quiescence and hematopoietic homeostasis.

The quiescence is essential for HSC to preserve its functional features, including self-renewal and lineage commitment potentials [15, 18]. In most cases, loss of quiescence leads to HSC exhaustion and defective repopulation. For instance, the cell cycle regulators p21^cip1/waf1^ and p57^Kip2^ have been shown to prevent HSC ablation by maintaining its quiescence [45, 46]. Consistently, our data support that Znhit1-mediated quiescence preserves HSC population and hematopoietic homeostasis. Interestingly, p18^ink4c^ knockout resulted in HSC proliferation with enhanced repopulation capacity [47]. The underlying mechanism of how quiescence-escaped HSCs behave distinctly attracts further studies.

Hematopoietic phenotypes of Znhit1 deficiency, including HSC quiescence disruption, HSC functional exhaustion, myeloid expansion, T-lymphoid expansion, and B-lymphoid contraction, are consistent with those of Pten loss [19, 20]. Moreover, inhibition of PI3K–Akt signaling by LY294002 efficiently suppresses acute expansion and followed exhaustion of HSCs and rescues B-lymphoid specification defect caused by Znhit1 deletion. These solidly demonstrate that Znhit1 controls HSC quiescence and B-lymphoid specification through Pten–PI3K–Akt pathway. Pten loss has also been closely related to leukemogenesis, which is represented by transition from normal HSCs to leukemia-initiating cells [48, 49]. Further experiments are needed to address whether Znhit1/H2A.Z participates in malignant hematopoiesis. Moreover, as we defined the critical roles of distal enhancer and its accessibility in regulating *Pten* transcription, it is important to extend the research to other organ tumorigenesis.

Ye et al. [50] reported that Znhit1 guided lymphoid differentiation of MPPs. We also observed myeloid expansion and B-lymphoid contraction in PB after Znhit1 deletion. However, we demonstrated that these changes were independent of altered myeloid or lymphoid progenitors in BM. In consistent with our results, Pten-mutant mice also showed PB granulocytosis but no alteration of CMP, GMP, or MEP in the BM [19, 20]. More importantly, our study established the essential function of Znhit1/H2A.Z in balancing HSC quiescence and exhaustion, which was not observed in their research. This difference should be due to the tools employed: we induced Znhit1 deletion in HSCs in vivo, while Ye et al. generated CRISPR-edited Znhit1-null HSCs in vitro, and then transplanted them into lethal-irradiation recipient mice. The long-term in vitro culture required for gene editing and followed selection might wipe off the primary effect of Znhit1 deficiency on HSC quiescence [51]. Besides, given that tissue stem cells are heterogeneous and contain undefined subpopulations, it is difficult to rule out the possibility that remaining transplanted HSCs are resistant to Znhit1 loss. These studies suggest that in vitro gene editing followed by transplantation might be inappropriate for investigation of HSC quiescence.

By employing genetic mice tools, our study pinpoints a dominant function of Znhit1/H2A.Z in regulating HSC quiescence, providing the first in vivo description of how Znhit1-mediated chromatin remodeling determines cell fate through controlling distal accessibility. Further investigation of how Znhit1/H2A.Z cooperates with histone modifications or/and DNA methylation to regulate transcriptome for tissue homeostasis are warranted.

## Supplementary information

Supplementary Material

## Data Availability

The NGS data generated in this study were deposited to the NCBI SRA database under accession number SRP201517 (RNA-seq data) and SRP201526 (ATAC-seq data).
