# Supplementary material for: Chromatin remodeler Znhit1 preserves hematopoietic stem cell quiescence by determining the accessibility of distal enhancers
Source: Leukemia. 2020 Jul 21;34(12):3348–58. doi: 10.1038/s41375-020-0988-5 (PMC7685981; doi:10.1038/s41375-020-0988-5)
Supplement: Supplementary file 1 — Supplementary Material [file 41375_2020_988_MOESM1_ESM.docx]

**Supplementary Information**

**Chromatin Remodeler Znhit1 Preserves Hematopoietic Stem Cell Quiescence by Determining the Accessibility of Distal Enhancers**

**Sun et al.**

**Supplementary Methods**

**Supplementary Figure 1-5**

**Supplementary Table 1**

**Supplementary Methods**

**Flow cytometry antibodies.** We employed FITC- or Pacific Blue-anti-Lineage cocktail (anti-CD3 (17A2), anti-Ly-6G/Ly-6C (RB68C5), anti-CD11b (M1/70), anti-CD45R/B220 (RA36B2) and anti-TER-119 (Ter-119)) for lineages; Alexa Fluor 647-anti-CD34 (SA376A4), APC-anti-CD48 (HM48-1), APC-Cy7-anti-CD117 (2B8), PE-anti-CD150 (TC15-12F12.2), PE-anti-Flt3 (A2F10), PE-Cy5-anti-CD127 (A7R34), PE-Cy7-anti-CD16/32 (93) and PerCP-Cy5.5-anti-Sca1 (D70) for HSCs, LMPPs, CMP, GMPs, MEPs and CLPs; PerCP-Cy5.5-anti-CD19 (1D3/CD19) and FITC-anti-B220 (RA3-6B2) for B lymphocytes; APC-Cy7-anti-Gr1 (RB6-8C5) and PerCP-Cy5.5-anti-CD11b (M1-70) for myeloid cells; PE-Cy7-anti-CD3ε (145-2C11) for T lymphocytes; APC- or FITC-anti-CD45.2 (104) and PE-anti-CD45.1 (A20) for chimeric ratio in BM transplantation.

**Colony-forming unit (CFU) assay.** 2.0×10^4^ spleen cells from control or *Znhit1*^-/-^ mice were cultured in MethoCult™ medium (STEMCELL Technologies, M3434) to detect multiple hematopoietic cell colonies including BFU-E (erythroid), CFU-GM (granulocyte, macrophage) and CFU-GEMM (granulocyte, erythrocyte, monocyte, macrophage).

**Competitive repopulation assay.** 1.0×10^6^ BM cells from *Znhit1*^fl/fl^; *Mx1-cre* mice or their littermate controls (CD45.2^+^) together with equal number of competitor BM cells (CD45.1^+^) were transplanted into lethally irradiated recipients (9.0 Gy, CD45.1^+^) through tail vein. To assess the contribution of donor cells (CD45.2^+^) to multi-lineages reconstitution, PB and BM cells were subjected to flow cytometry.

**Immunofluorescence.** Different blood cells (LT-HSCs, ST-HSCs, MPPs, T cells, B cells, Monocytes and Granulocytes) from 8-week-old C57BL/6 mice were sorted and deposited on slides using Cytospin for anti-Znhit1 (Sino Biological-203239-T38) immunostaining. The signals were examined by using confocal laser scanning microscope (FV3000, Olympus, Japan) and quantifications of fluorescent intensity were obtained by using ImageJ (Version 1.52a).

**Histology.** For morphological analysis, 5 μm spleen sections were prepared for hematoxylin eosin staining. Images were taken by Vectra Automated Quantitative Pathology Imaging System (Perkin Elmer).

**RNA-seq.** RNA from freshly sorted Lin^-^Sca1^+^cKit^+^ cells (LSKs) (1.0×10^4^) was converted into cDNA library then subjected to high-throughput sequencing. Over 40 million reads were obtained per sample on Illumina HiSeq platform for 3 biological replicates. The RNA-seq data was mapped to mm10 genome by TopHat v2.0.8 [1] with no more than 2 mismatches, and only the uniquely mapped reads were used to estimate the expression values in gene level by RPKM [2]. Statistical significant test of differentially expressed genes was performed by DEseq with R. Genes with absolute log2-transformed fold changes greater than 1.8 were regarded as differentially expressed genes and a threshold of p-value < 0.05 was used. Hierarchical clustering of log2-transformed RPKMs was generated by Cluster 3.0 and visualized by Java TreeView. Gene set enrichment analysis was performed with GSEA v3.0 software (available from the Broad Institute) [3].

**ATAC-seq.** Freshly sorted LSK cells (1.0×10^4^) were subjected to lysis buffer (10 mM Tris-HCl (pH 7.4), 10 mM NaCl, 3 mM MgCl_2_, 0.5% NP-40) for 10 min on ice, then spun at 500 g for 5 min to obtain nuclei pellet [4]. Nuclei were then segmented with Tn5 transposase (Vazyme Biotech) at 37℃ for 30 mins. Constructed library was sequenced on Illumina HiSeq X Ten by BerryGenomics for 2 biological replicates. ATAC-seq reads were aligned to the reference genome (mm10) using Bowtie2 v2.3.3.1 with no more than 2 mismatches. All non-uniquely mapped reads and reads mapped to mitochondria sequence were removed. Only the uniquely mapped reads were used for peak calling analysis. The peaks detection was performed by MACS with default cutoff. Peaks were assigned to the nearest genes using ChIPseeker with R. HOMER was performed for motif analysis [5]. ChIP-seq data of H3K4me1, H3K4me3 and H3K27ac in HSC are reported by Matthias group [6].

**Quantitative RT-PCR (qRT-PCR).** This assay was performed as previously described [7] . Primers used were listed as follows.

| Primers | Forward (5’-3’) | Reverse (5’-3’) |
| --- | --- | --- |
| *H3* | TGTGGCCCTCCGTGAAAT C | GGCATAATTGTTACACGTTTGGC |
| *Znhit1* | TGGGCAAGAGGCTACCTC A | CAGATGCACTCAGGTTCTGCT |
| *H2afz* | CCAAGACAAAGGCGGTTTCC | TCCTGCCAACTCAAGTACCTC |
| *H2afv* | GCTAAGGCGGTGTCTCGTTC | TGTGGTGCGAGTCTTCAAGTG |
| *Pten* | TTTGCTAGTGAGTGGAATCCTCT | TGTGACAAAAGTGACACAGATCA |
| *Egr1* | TATGAGCACCTGACCACAGAG | GCTGGGATAACTCGTCTCCA |
| *Klf4* | GGCGAGTCTGACATGGCTG | GCTGGACGCAGTGTCTTCTC |
| *Foxo1* | CCCAGGCCGGAGTTTAACC | GTTGCTCATAAAGTCGGTGCT |
| *Fstl1* | CACGGCGAGGAGGAACCTA | TCTTGCCATTACTGCCACACA |
| *Mpl* | CGGGTGCTGTTTGTGGATAGT | GGAAGTCACTGATTTCAGGAGC |
| *Nr4a1* | TTGAGTTCGGCAAGCCTACC | GTGTACCCGTCCATGAAGGTG |

**CRISPR/Cas9 targeting**. Paired sgRNAs (sgRNA_1: 5’- ACCATTATGGGTGACAC TCTTGG -3’, sgRNA_2: 5’- CAGGTAACCCAGTTCTCCATGGG -3’) were cloned individually into pSpCas9(BB)-2A-GFP (PX458), pSpCas9 (BB)-2A-Puro (PX459) or lentiCRISPRv2 plasmids [8] for *Pten* enhancer deletion. psPAX2 , pMD2.G and lentiCRISPRv2 were used to product lentivirus. The EML cells were transfected by lentiCRISPRv2 containing sgRNA lentivirus, and GFP^+^ cells were sorted for culture 48 hours then analyzed by using qRT-PCR. The genomic DNA was extracted for genotyping with primer pair: sense 5’- CAAACCACCATAACATACATGC -3’, anti-sense 5’- GGAAAAGGGAAAACCTCAAA -3’.

**References**

1. Trapnell C, Pachter L, Salzberg SL. TopHat: discovering splice junctions with RNA-Seq. Bioinformatics. 2009;25:1105-1111.

2. Mortazavi A, Williams BA, McCue K, Schaeffer L, Wold B. Mapping and quantifying mammalian transcriptomes by RNA-Seq. Nat Methods. 2008;5:621-628.

3. Subramanian A, Tamayo P, Mootha VK, Mukherjee S, Ebert BL, Gillette MA, et al. Gene set enrichment analysis: a knowledge-based approach for interpreting genome-wide expression profiles. Proc Natl Acad Sci U S A. 2005;102:15545-15550.

4. Buenrostro JD, Giresi PG, Zaba LC, Chang HY, Greenleaf WJ. Transposition of native chromatin for fast and sensitive epigenomic profiling of open chromatin, DNA-binding proteins and nucleosome position. Nat Methods. 2013;10:1213-1218.

5. Heinz S, Benner C, Spann N, Bertolino E, Lin YC, Laslo P, et al. Simple combinations of lineage-determining transcription factors prime cis-regulatory elements required for macrophage and B cell identities. Mol Cell. 2010;38:576-589.

6. Choukrallah MA, Song S, Rolink AG, Burger L, Matthias P. Enhancer repertoires are reshaped independently of early priming and heterochromatin dynamics during B cell differentiation. Nat Commun. 2015;6:8324.

7. Zhao B, Qi Z, Li Y, Wang C, Fu W, Chen YG. The non-muscle-myosin-II heavy chain Myh9 mediates colitis-induced epithelium injury by restricting Lgr5+ stem cells. Nat Commun. 2015;6:7166.

8. Ran FA, Hsu PD, Wright J, Agarwala V, Scott DA, Zhang F. Genome engineering using the CRISPR-Cas9 system. Nature Protocols. 2013;8:2281-2308.

**Supplementary Figure 1**

**
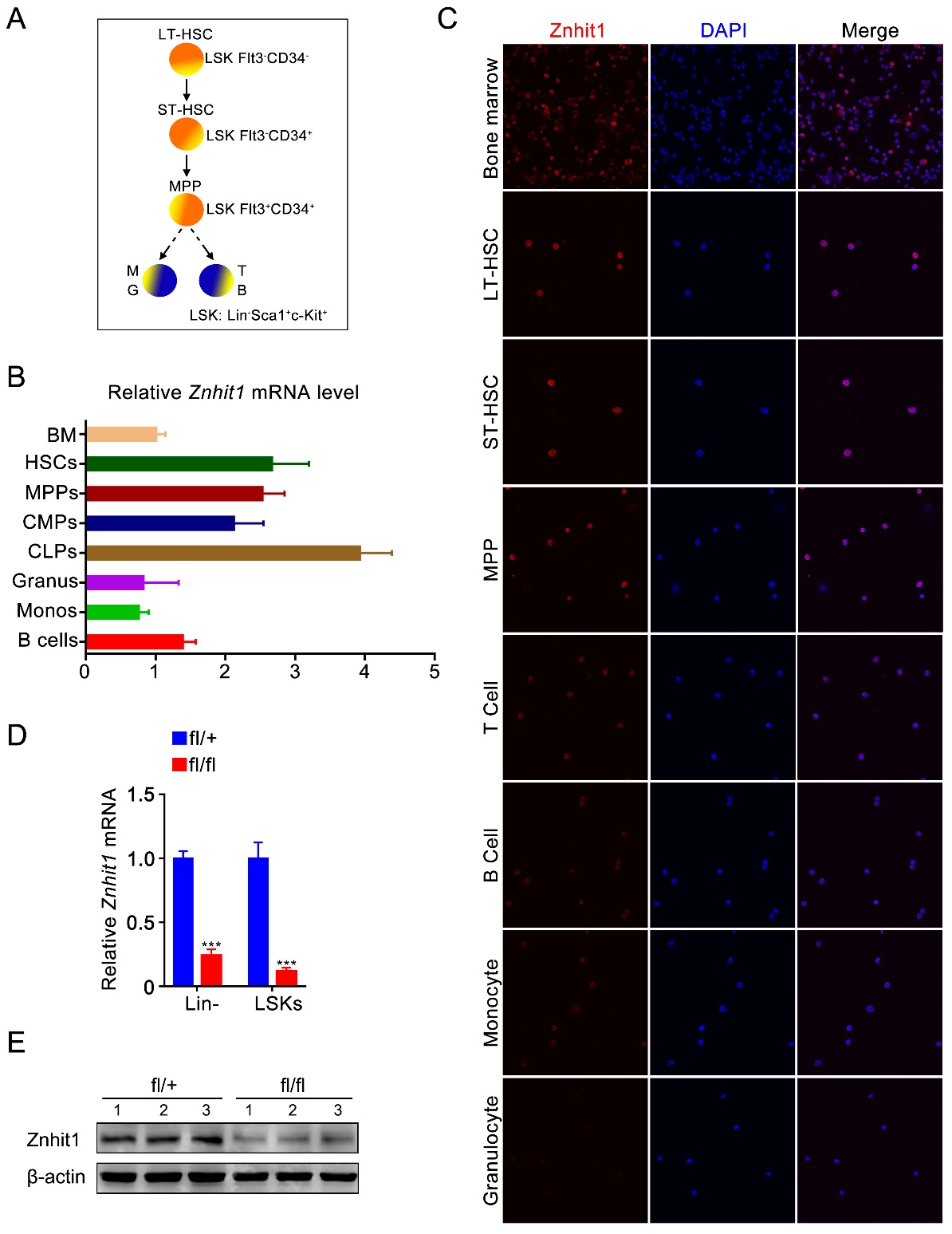
**

**Supplementary Figure 1∣Hematopoietic deletion of Znhit1 with *Mx1-cre*.** (**A and B**) Hematopoietic lineages were sorted from adult mice to examine the expression of *Znhit1* using qRT-PCR. For qRT-PCR, histone H3 was used as an internal control. (**C**) Hematopoietic lineages were sorted from adult mice and deposited on slides using Cytospin for anti-Znhit1 immunostaining to examine the protein level of Znhit1. (**D**) qRT-PCR was performed to show *Znhit1* expression in Lin^-^ cells and LSKs from control (fl/+) and *Znhit1*^-/-^ (fl/fl) mice. (**E**) Western blotting of Znhit1 in Lin^-^ cells from control (fl/+) and *Znhit1*^-/-^ (fl/fl) mice. β-actin was used as an internal control. Results were representative of at least three independent experiments. Data were presented as mean±s.d. ******* indicates *p*<0.001.

**Supplementary Figure 2**

**
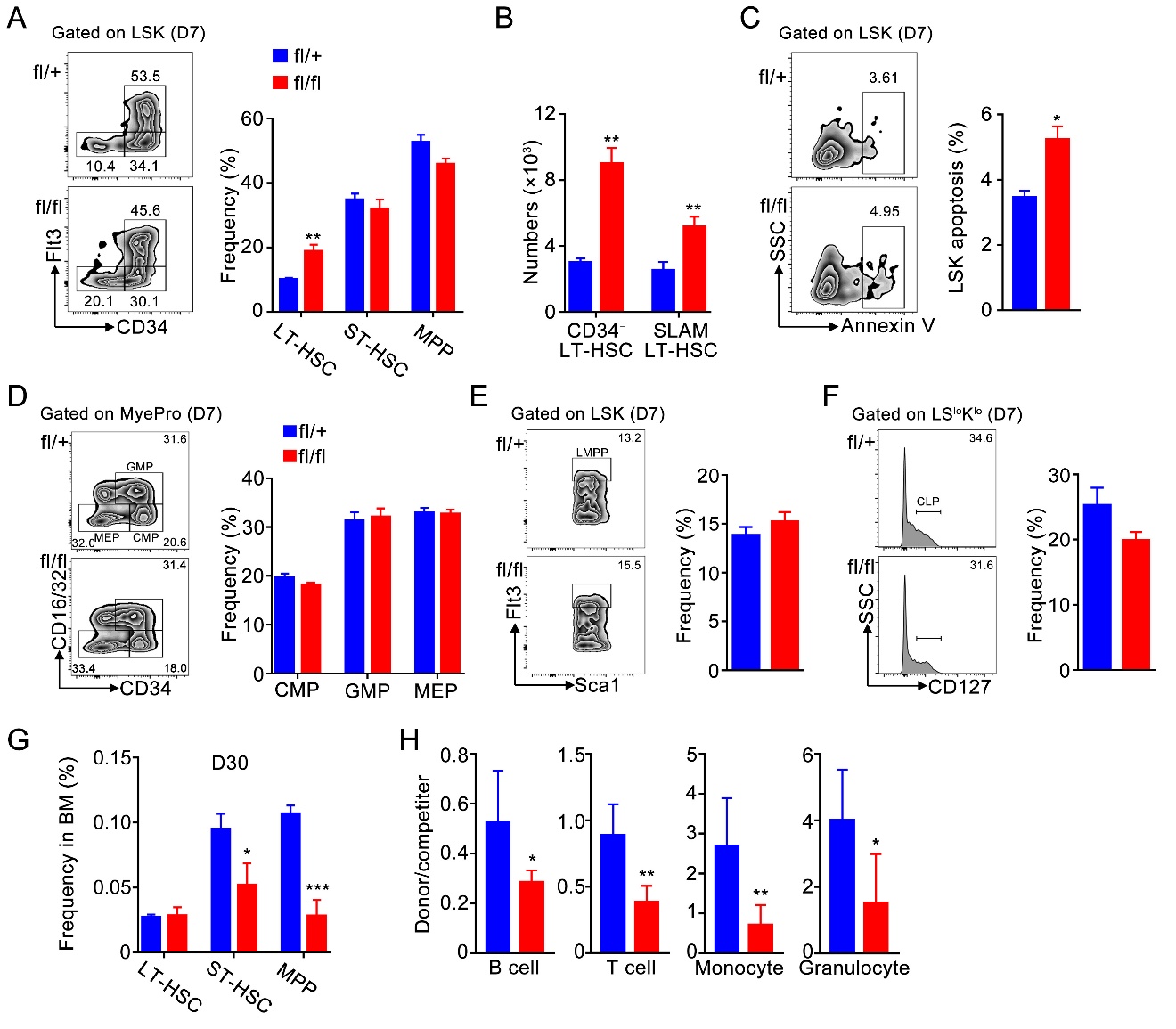
**

**Supplementary Figure 2∣Loss of Znhit1 impairs hematopoietic functions.** (**A**) Flow cytometry of BM cells showed the gates and frequencies of CD34^-^ LT-HSC, ST-HSC and MPP from control (fl/+) and *Znhit1*^-/-^ (fl/fl) mice at D7 following pIpC treatment (*n*=3). (**B**) Flow cytometry of BM cells showed the gates and frequencies of CMP, GMP and MEP from control (fl/+) and *Znhit1*^-/-^ (fl/fl) mice at D7 following pIpC treatment (*n*=3). (**C**) Total cells of CD34^-^ LT-HSC and SLAM LT-HSC in BM from control (fl/+) and *Znhit1*^-/-^ (fl/fl) mice at D7 following pIpC treatment (*n*=3). (**D-F**) Flow cytometry of BM cells showed the gates and frequencies of myeloid progenitors (**D**), LMPP (**E**), and CLP (**F**) from control (fl/+) and *Znhit1*^-/-^ (fl/fl) mice at D7 following pIpC treatment (*n*=3). (**G**) The frequencies of LT-HSC, ST-HSC and MPP in the BM cells were calculated from control (fl/+) and *Znhit1*^-/-^ (fl/fl) mice at D30 (day 30) following pIpC treatment (*n*=3). (**H**) Analysis of the donor (CD45.2^+^) contribution to B cell, T cell, monocyte, and granulocyte 4 months following competitive BM transplantation (*n*=5). Data were presented as mean±s.d. ***** indicates *p*＜0.05; ****** indicates *p*＜0.01; ******* indicates *p*＜0.001.

**Supplementary Figure 3**

**
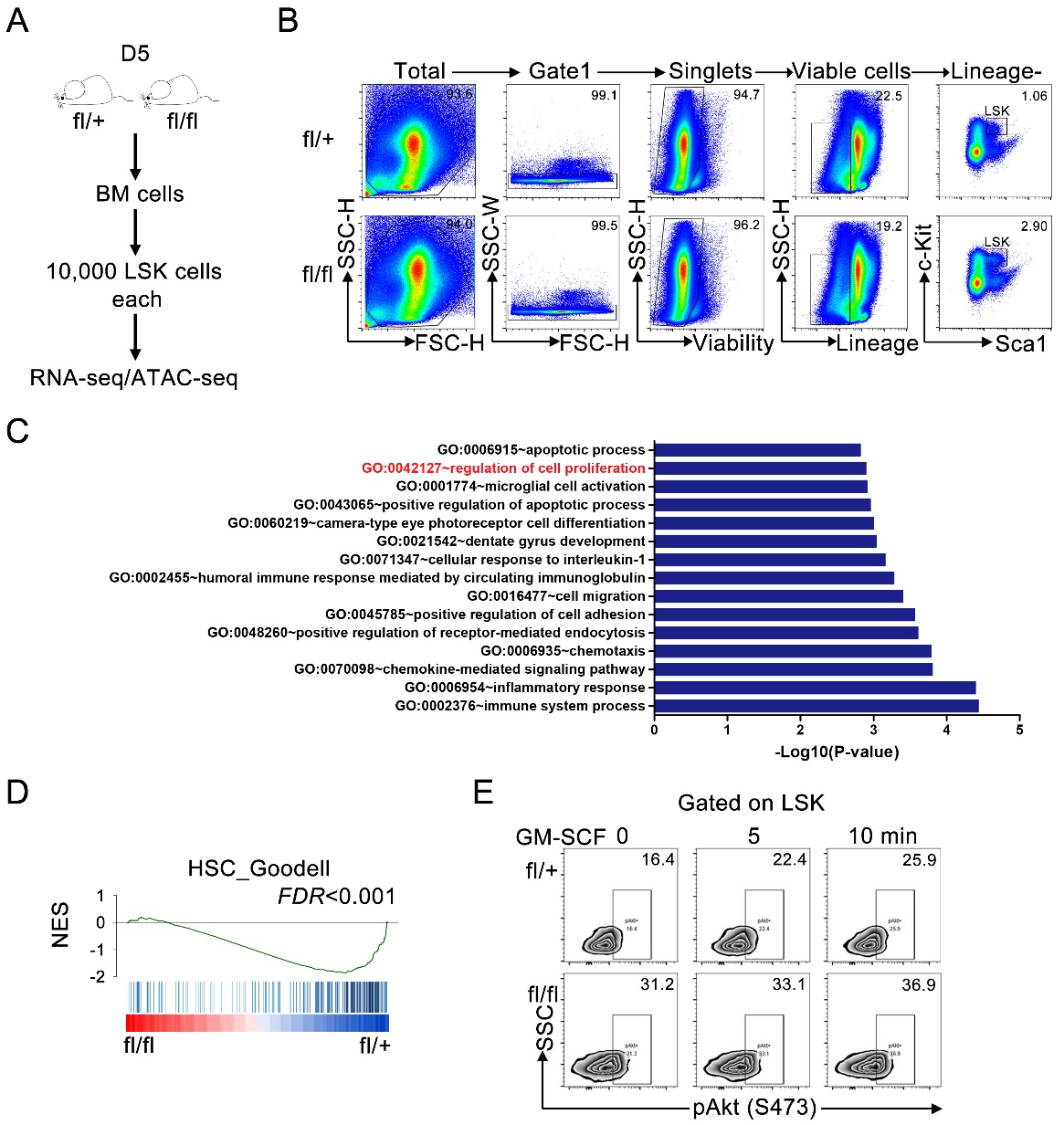
**

**Supplementary Figure 3∣Znhit1 regulates HSC quiescence through PI3K/Akt signaling.** (**A**) RNA-seq and ATAC-seq workflow in control (fl/+) and *Znhit1*^-/-^ (fl/fl) LSKs at D5 (day 5) following pIpC treatment. (**B**) Representative plots for sorting LSKs in BM from control (fl/+) and *Znhit1*^-/-^ (fl/fl) mice at D5 following pIpC treatment. (**C**) Gene Ontology (GO) analysis highlighted the changed biological processes in LSKs after Znhit1 deletion. Red denote the term of regulation of cell cycle. (**D**) Gene set enrichment analysis (GSEA) of selected gene sets encoding products related to HSC signature by Goodell, presented as normalized enrichment score (NES). Gene expression data come from control (fl/+) and *Znhit1*^-/-^ (fl/fl) LSKs. (**E**) Flow cytometry of the gates of Akt-phosphorylated (Ser473) in control (fl/+) and *Znhit1*^-/-^ (fl/fl) LSKs untreated or stimulated with GM-CSF for 5 or 10 minutes (*n*=3).

**Supplementary Figure 4**

**
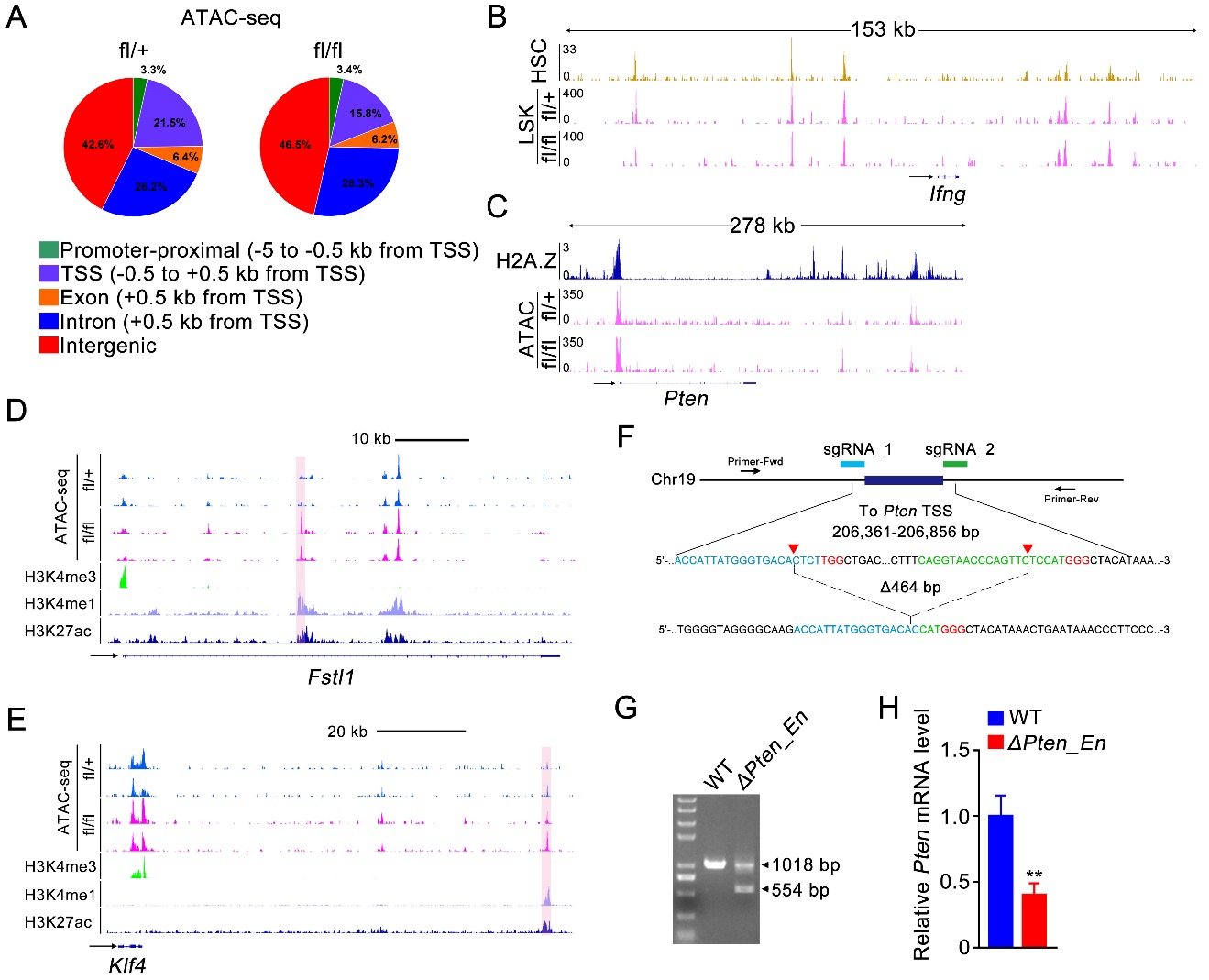
**

**Supplementary Figure 4∣Znhit1 modulates chromatin accessibility for gene expression.** (**A**) The genomic distribution of accessible ATAC peaks of LSKs from control (fl/+) and *Znhit1*^-/-^ (fl/fl) mice at D5 following pIpC treatment. (**B**) Example ATAC-seq tracks for *Ifng* locus in HSC (Shih HY, et al. 2016) and LSKs show comparable signal-to-noise ratio. (**C**) Example tracks of ATAC-seq and H2A.Z ChIP-seq (Kovalchuk AL, et al. 2012) for *Pten* locus show collaborative regulation in distal intergenic regions. (**D and E**) ATAC-seq signals and ChIP-seq signals for H3K4me3, H3K4me1 and H3K27ac binding at *Fstl1* (**D**) and *Klf4* (**E**) loci, and ChIP-seq data come from published data (see methods). (**F**) Paired sgRNAs are designed to excise the *Pten* distal enhancer (+206,361 to +206,856). Target sequences are shown in respective colors, and the cleavage sites are indicated by red triangles. (**G and H**) Deletion of distal enhancer down-regulated Pten expression in NIH3T3 cells. For qRT-PCR, H3 was used as an internal control. Results were representative of at least three independent experiments. Data were presented as mean±s.d. ****** indicates *p*<0.01.

**Supplementary Figure 5**

**
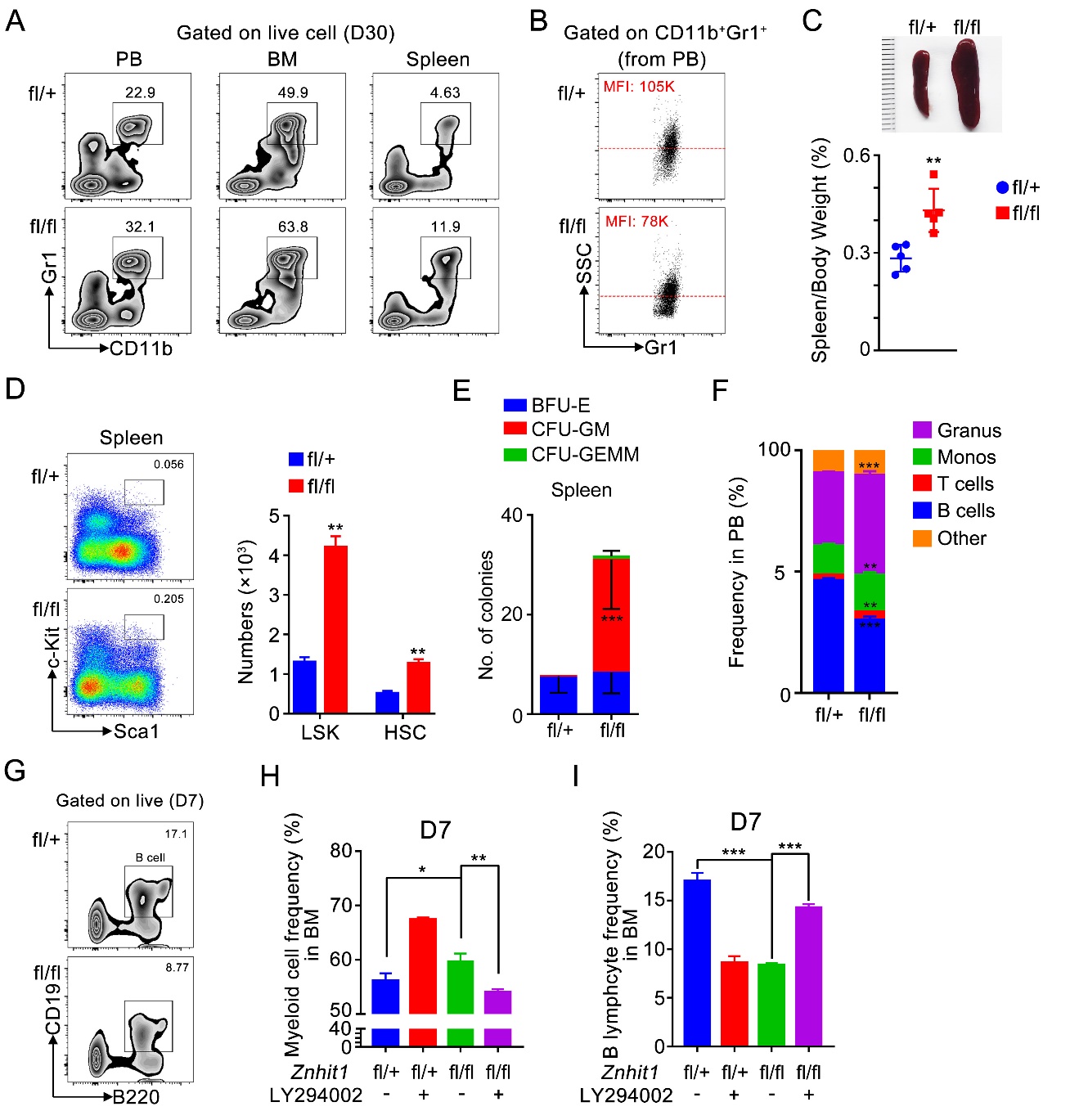
**

**Supplementary Figure 5∣Znhit1-Pten-PI3K-Akt axis controls myeloid expansion and B-lymphoid specification.** (**A**) Flow cytometry of PB, BM and spleen cells showed the gate and frequency of myeloid cells from control (fl/+) and *Znhit1*^-/-^ (fl/fl) mice at D30 following pIpC treatment (*n*=3). (**B**) The SSC MFI of PB myeloid cells from control (fl/+) and *Znhit1*^-/-^ (fl/fl) mice. (**C**) Spleen/body weight ratio of control (fl/+) and *Znhit1*^-/-^ (fl/fl) mice at D30 following pIpC treatment (*n*=5). (**D**) Flow cytometry of spleen cells showed the gate, frequency and numbers of LSKs and HSCs from control (fl/+) and *Znhit1*^-/-^ (fl/fl) mice at D7 following pIpC treatment. (**E**) CFU assay of spleen cells from control (fl/+) and *Znhit1*^-/-^ (fl/fl) mice at D7 following pIpC treatment. (**F**) Flow cytometry of PB showed the frequencies of granulocytes, monocytes, T cells and B cells from control (fl/+) and *Znhit1*^-/-^ (fl/fl) mice at D30 following pIpC treatment (*n*=3). (**G**) Flow cytometry of BM cells showed the gate of B lymphocytes from control (fl/+) and *Znhit1*^-/-^ (fl/fl) mice at D7 following pIpC treatment (*n*=3). (**H and I**) At day 7 following Znhit1 deletion, flow cytometry of BM cells showed the frequency of control (fl/+) and *Znhit1*^-/-^ (fl/fl) myeloid cells (**H**) or B lymphocytes (**I**) with/without daily LY294002 treatment for total 7 days after pIpC injection (*n*=3). Data were presented as mean±s.d. ***** indicates *p*＜0.05; ****** indicates *p*＜0.01; ******* indicates *p*＜0.001.

**Supplementary Table 1∣415 Znhit1-regulated genes with changed ATAC-seq peaks.**

| Gene Symbol | log2FoldChange | | *P*-value |
| --- | --- | --- | --- |
| Dusp1 | -1.58045 | 1.23E-16 | |
| Egr1 | -2.43454 | 5.24E-16 | |
| Klf6 | -1.60688 | 2.04E-14 | |
| Rgs1 | -1.93689 | 4.92E-13 | |
| Itgb7 | 1.61226 | 7.36E-13 | |
| Cd48 | 1.032193 | 4.24E-10 | |
| Sell | 1.063813 | 2.36E-09 | |
| Rab7b | 2.201255 | 5.87E-09 | |
| Krt18 | -1.09547 | 6.55E-09 | |
| Cdh15 | -9.84298 | 1.76E-08 | |
| Ccl3 | -1.00382 | 4.28E-08 | |
| Ier2 | -1.07861 | 2.6E-07 | |
| Emcn | -1.91037 | 3.01E-07 | |
| Ptprk | 8.67455 | 3.66E-07 | |
| Spns3 | 0.917104 | 6.73E-07 | |
| Igf1 | -3.51064 | 7.88E-07 | |
| Dhrs1 | -0.99743 | 8.12E-07 | |
| Fosl1 | -9.2808 | 1.93E-06 | |
| Hacd4 | -1.56419 | 4.17E-06 | |
| Smpdl3a | -0.90988 | 4.24E-06 | |
| Hsf2bp | 9.033851 | 4.59E-06 | |
| Ldhb | -1.02869 | 9.31E-06 | |
| Syne1 | -8.61454 | 1.34E-05 | |
| Nfia | -1.10671 | 1.49E-05 | |
| Il10 | -6.68428 | 1.69E-05 | |
| Cd83 | -8.48493 | 1.85E-05 | |
| Zfp618 | -3.63926 | 2.42E-05 | |
| Nfkbiz | -1.16372 | 2.49E-05 | |
| Sstr2 | 1.344786 | 2.82E-05 | |
| Mogat2 | 8.906292 | 2.86E-05 | |
| Tmem204 | 9.160914 | 2.96E-05 | |
| Ptges | -3.90606 | 3.2E-05 | |
| Cpq | -0.88483 | 3.71E-05 | |
| Clec14a | 7.604044 | 4.08E-05 | |
| Lrrn3 | 8.203267 | 4.1E-05 | |
| Slc31a2 | -1.96542 | 4.14E-05 | |
| Maged1 | -1.09975 | 4.46E-05 | |
| Pmaip1 | 5.200506 | 4.72E-05 | |
| Emilin2 | 1.541965 | 5.59E-05 | |
| Car5b | -8.20958 | 5.96E-05 | |
| Cmah | 2.51185 | 6.09E-05 | |
| Cd2 | -8.45338 | 6.17E-05 | |
| Arl3 | -1.85714 | 6.2E-05 | |
| Pea15a | -1.10578 | 6.35E-05 | |
| Gpr174 | 7.487404 | 6.48E-05 | |
| Pten | -0.94256 | 7.1E-05 | |
| 5730409E04Rik | -1.81193 | 8.79E-05 | |
| Steap3 | 2.168665 | 8.94E-05 | |
| Ly6i | 8.445301 | 9.33E-05 | |
| Ppp1r9a | -1.6587 | 9.42E-05 | |
| 2010300C02Rik | 7.370944 | 0.00011 | |
| Pkia | -6.48492 | 0.000112 | |
| Atp10a | 1.487761 | 0.000117 | |
| Fstl1 | -1.49638 | 0.000129 | |
| Ampd3 | -2.49753 | 0.000129 | |
| Depdc1b | 1.846023 | 0.000132 | |
| Mb21d2 | -3.74717 | 0.000133 | |
| Pde1c | -6.93558 | 0.000133 | |
| Camk2b | 2.262416 | 0.000146 | |
| Jun | -0.94033 | 0.000154 | |
| Fuk | -3.14353 | 0.000155 | |
| Dram1 | 5.720786 | 0.000155 | |
| Rapgef4 | -8.00044 | 0.000167 | |
| Pstpip2 | -5.75195 | 0.000174 | |
| Nrg1 | 7.433736 | 0.000176 | |
| Galm | -1.62907 | 0.000178 | |
| Runx1t1 | -7.81169 | 0.000194 | |
| Mpeg1 | 1.270235 | 0.000198 | |
| Fhl2 | 7.849699 | 0.000203 | |
| Mtap | 0.942145 | 0.000277 | |
| Skil | -1.07727 | 0.000278 | |
| Ncam1 | 7.47514 | 0.00028 | |
| Pkib | 1.352455 | 0.000299 | |
| 0610040J01Rik | -6.28495 | 0.000304 | |
| Clip3 | -4.29873 | 0.000317 | |
| Plxdc2 | -1.96423 | 0.000341 | |
| Ccl9 | 1.103765 | 0.000346 | |
| Slc4a8 | 1.597162 | 0.000355 | |
| Camk1 | -0.87269 | 0.000367 | |
| Cnn3 | 1.372508 | 0.000373 | |
| Rffl | -1.26039 | 0.000375 | |
| Sik1 | -2.07247 | 0.000397 | |
| Mefv | 6.86295 | 0.000424 | |
| Tgm2 | -1.29243 | 0.000428 | |
| Wdr60 | -1.47791 | 0.000432 | |
| Rbp1 | -0.94628 | 0.000453 | |
| Paqr3 | -4.73156 | 0.00052 | |
| Plekha7 | 3.674359 | 0.000533 | |
| Lgmn | 1.295162 | 0.000559 | |
| Fubp3 | -1.11688 | 0.000578 | |
| Ccne1 | 1.487815 | 0.000589 | |
| Ear1 | -6.15701 | 0.000613 | |
| Gm14085 | 7.417613 | 0.000616 | |
| Akr1e1 | -0.98238 | 0.00062 | |
| Sorbs3 | -0.84834 | 0.000627 | |
| Ms4a4b | 2.584085 | 0.000667 | |
| Nr3c2 | -6.34731 | 0.000692 | |
| Elmod3 | -1.39704 | 0.0007 | |
| Klf2 | -1.68528 | 0.000718 | |
| Gadd45gip1 | 1.030841 | 0.000779 | |
| Cstb | -0.99129 | 0.000793 | |
| Ass1 | 1.065404 | 0.000814 | |
| Bcar3 | 7.538 | 0.000888 | |
| P3h4 | -6.04436 | 0.000901 | |
| Prps1 | 0.876673 | 0.000904 | |
| Lrrc9 | -6.69962 | 0.000942 | |
| Mecom | -0.95046 | 0.000982 | |
| Cst7 | 1.14045 | 0.001001 | |
| Ryk | -2.53102 | 0.001021 | |
| Shisa2 | 8.533545 | 0.001034 | |
| Myo1e | 5.072064 | 0.001037 | |
| Gpr150 | -7.59604 | 0.001064 | |
| Pex26 | -1.49052 | 0.001095 | |
| Drc7 | 1.263774 | 0.001107 | |
| Adamts3 | 1.595687 | 0.001126 | |
| Iqck | 7.424898 | 0.001131 | |
| D930020B18Rik | 2.856358 | 0.001208 | |
| Slc16a12 | -4.64323 | 0.001476 | |
| Gna14 | 3.137707 | 0.001523 | |
| Ttc38 | -1.07419 | 0.001539 | |
| Dus2 | -1.39874 | 0.001559 | |
| 1110032A03Rik | -1.32394 | 0.001573 | |
| Tyro3 | -6.81858 | 0.001659 | |
| Btbd16 | -7.32575 | 0.001686 | |
| Parm1 | 4.779846 | 0.001701 | |
| Mrvi1 | 3.02962 | 0.00171 | |
| Trib1 | -1.46474 | 0.001718 | |
| Cd33 | 1.367389 | 0.001761 | |
| Ccl2 | 4.983562 | 0.0018 | |
| Epx | -3.03873 | 0.001809 | |
| Ern1 | -1.05946 | 0.001825 | |
| Spry2 | -0.92175 | 0.001867 | |
| Ggh | -1.27164 | 0.001879 | |
| Nsun7 | -4.77319 | 0.001896 | |
| Numb | -1.6819 | 0.001927 | |
| Slc22a23 | 6.211352 | 0.001976 | |
| Myof | -1.19977 | 0.002007 | |
| Bok | 0.973911 | 0.002099 | |
| Ak4 | 1.602623 | 0.002142 | |
| Abca13 | 7.726398 | 0.002167 | |
| Ccl5 | 1.213297 | 0.002321 | |
| Ttll7 | -5.6473 | 0.002385 | |
| Hectd3 | -1.3693 | 0.002401 | |
| Fndc7 | 8.418593 | 0.002444 | |
| Ccnb2 | 1.009113 | 0.002451 | |
| Zfp882 | -3.62985 | 0.002558 | |
| Sipa1l1 | 0.870853 | 0.002622 | |
| Map9 | -2.15806 | 0.002716 | |
| Hoxa4 | -7.35672 | 0.002789 | |
| Cnr1 | -7.33443 | 0.002912 | |
| Shisa9 | -8.21533 | 0.003003 | |
| Rnls | -2.04978 | 0.003067 | |
| Arhgap11a | 0.88597 | 0.003217 | |
| Rufy4 | 2.967725 | 0.003326 | |
| D3Ertd254e | -1.53679 | 0.003369 | |
| Pcyt1b | -3.11217 | 0.003406 | |
| 8430408G22Rik | 1.35316 | 0.003606 | |
| S1pr3 | 2.591962 | 0.003803 | |
| Rilpl1 | -1.42204 | 0.003808 | |
| Ptprf | 2.707374 | 0.003811 | |
| Clnk | 1.398606 | 0.003828 | |
| Espl1 | 1.07672 | 0.003841 | |
| Lpar6 | -1.30602 | 0.003888 | |
| Dgat2 | 4.351685 | 0.003976 | |
| Ago4 | -1.435 | 0.004041 | |
| Ctdp1 | 0.984184 | 0.004144 | |
| Aldh1b1 | -1.01362 | 0.004239 | |
| Fos | -1.8024 | 0.00428 | |
| Magi2 | -7.68926 | 0.004401 | |
| Wfs1 | -2.37127 | 0.004417 | |
| Krba1 | -1.2491 | 0.004449 | |
| Tmem141 | -1.48344 | 0.004567 | |
| 1700010I14Rik | -3.72029 | 0.004629 | |
| Sort1 | -0.87723 | 0.004639 | |
| Wwox | -1.01133 | 0.004666 | |
| Slc26a9 | -5.3184 | 0.004669 | |
| Gdpd5 | 5.58248 | 0.004672 | |
| Haus6 | 0.860325 | 0.004758 | |
| Adgre1 | 5.865911 | 0.004787 | |
| Gcc1 | -1.97162 | 0.004788 | |
| Gpr137c | -7.05945 | 0.004794 | |
| Fbn1 | -4.34481 | 0.00508 | |
| Mmrn1 | -1.09098 | 0.005272 | |
| Fcgr4 | 3.562831 | 0.005307 | |
| Zfp532 | -1.63499 | 0.005324 | |
| Dusp10 | 2.307943 | 0.005441 | |
| Srxn1 | -1.79261 | 0.005587 | |
| Noa1 | 1.011323 | 0.005596 | |
| Rnf32 | -6.31624 | 0.005683 | |
| Fbln1 | 4.101725 | 0.005811 | |
| Cd244 | 1.207715 | 0.005813 | |
| Rnf149 | 1.067288 | 0.005845 | |
| Tbrg4 | 0.944273 | 0.005851 | |
| Klf4 | -2.34348 | 0.005876 | |
| Acp2 | -1.04552 | 0.005961 | |
| Slc30a5 | 1.027936 | 0.005968 | |
| Cebpe | 2.721212 | 0.006061 | |
| Lysmd4 | -3.67455 | 0.006076 | |
| Naa25 | 0.955218 | 0.006192 | |
| Fgd4 | -2.11363 | 0.006222 | |
| Trim5 | -1.28353 | 0.006535 | |
| Klf12 | -3.13454 | 0.006546 | |
| Ncoa6 | -1.15578 | 0.006644 | |
| Ctu1 | -1.07693 | 0.006837 | |
| Akap1 | 1.123385 | 0.006927 | |
| Mfsd6 | -0.88859 | 0.006987 | |
| Slc16a2 | -2.24866 | 0.007028 | |
| Dock6 | -1.98313 | 0.007044 | |
| Syn3 | -2.83719 | 0.007237 | |
| Egln3 | 1.048595 | 0.007267 | |
| Mterf1b | 5.95356 | 0.007342 | |
| Bend5 | -1.2792 | 0.007637 | |
| S1pr1 | -1.28841 | 0.007706 | |
| Mbip | -0.86845 | 0.007721 | |
| Mtfp1 | 1.233389 | 0.007755 | |
| Cdh23 | -4.38162 | 0.007879 | |
| Sncaip | -8.38414 | 0.008055 | |
| Hormad2 | -3.91394 | 0.008461 | |
| Rtn4r | 2.614655 | 0.008552 | |
| Adgrl2 | -3.05948 | 0.008804 | |
| Plbd1 | -1.54798 | 0.00884 | |
| Nlrp3 | 6.955958 | 0.008968 | |
| Cd6 | 1.71131 | 0.00898 | |
| Flt1 | 6.769718 | 0.009083 | |
| Wdr7 | -1.92783 | 0.009144 | |
| Ly6c1 | 3.269671 | 0.009397 | |
| Klhl30 | 3.40946 | 0.00956 | |
| Fam110c | -3.52247 | 0.009571 | |
| 1110051M20Rik | -1.17456 | 0.00972 | |
| Cntnap1 | 4.173565 | 0.009811 | |
| Id2 | 0.90393 | 0.009947 | |
| Ccser2 | -1.32229 | 0.01004 | |
| Slc16a6 | 1.038829 | 0.010199 | |
| Klrb1f | 1.545623 | 0.010268 | |
| Tmod1 | 5.826083 | 0.01034 | |
| Poll | -0.86923 | 0.010401 | |
| Ccdc114 | -1.95365 | 0.010917 | |
| Mtmr11 | 4.411738 | 0.011064 | |
| Ppp1r26 | -1.69526 | 0.011117 | |
| Dhx32 | -1.18279 | 0.01124 | |
| 5830473C10Rik | 7.219529 | 0.011325 | |
| Hhat | -1.11498 | 0.011437 | |
| Elp6 | -1.1234 | 0.011565 | |
| Mmp2 | -1.3091 | 0.01159 | |
| Ccnd1 | 0.878515 | 0.011728 | |
| Cxcr6 | 3.590352 | 0.012022 | |
| Kcng1 | 1.140913 | 0.012097 | |
| Lmo7 | -4.37854 | 0.012123 | |
| Tspyl4 | -3.18042 | 0.012354 | |
| Spsb4 | 2.587042 | 0.012682 | |
| Ppp6r2 | -3.37782 | 0.012693 | |
| Il1rl2 | -4.29615 | 0.012809 | |
| Mctp1 | -0.96986 | 0.01286 | |
| 1110034G24Rik | -1.2648 | 0.013517 | |
| Prdm5 | -1.21495 | 0.013662 | |
| Cep164 | -0.89351 | 0.013699 | |
| Mageh1 | -1.33488 | 0.013855 | |
| Cdk20 | -2.2145 | 0.014083 | |
| Manba | -1.16383 | 0.014231 | |
| Lifr | 2.32133 | 0.014412 | |
| Lpcat2 | 1.582745 | 0.015137 | |
| Clec5a | 3.527286 | 0.015286 | |
| Arap2 | 1.145385 | 0.015572 | |
| Epas1 | -4.50015 | 0.015586 | |
| Adgrg5 | 2.727308 | 0.015591 | |
| Steap4 | 4.458096 | 0.015615 | |
| Pomk | -1.23031 | 0.01565 | |
| Gimap4 | 0.986853 | 0.015708 | |
| Fra10ac1 | -1.04701 | 0.016163 | |
| Slc41a3 | -1.32365 | 0.01632 | |
| Rps6kc1 | -1.1849 | 0.016366 | |
| Ldlrad4 | 1.014751 | 0.01656 | |
| Cdk14 | -2.27317 | 0.016783 | |
| Creb3l1 | -3.55284 | 0.017055 | |
| Hip1 | -1.00139 | 0.01722 | |
| Dtx4 | 1.242252 | 0.017287 | |
| Hivep1 | -0.91357 | 0.017443 | |
| Capsl | -2.04186 | 0.017598 | |
| Bhlhe40 | 2.814599 | 0.017811 | |
| Igf2bp2 | -2.16167 | 0.017966 | |
| Cep95 | -1.15803 | 0.018041 | |
| Prss57 | 0.923644 | 0.018219 | |
| Cand2 | -0.92649 | 0.018915 | |
| Cd7 | 0.960996 | 0.01897 | |
| Gadd45g | 1.763377 | 0.019051 | |
| Sult1a1 | -1.55474 | 0.019473 | |
| Neurl1a | 4.902589 | 0.019536 | |
| Hif3a | -1.30244 | 0.019715 | |
| Ctnnd1 | -0.86467 | 0.019862 | |
| Cnst | -1.40543 | 0.01991 | |
| Foxo1 | -1.07438 | 0.020004 | |
| Cxcl2 | -3.45838 | 0.020168 | |
| Map10 | -2.71989 | 0.020395 | |
| Clmn | -4.2784 | 0.020643 | |
| Tagap | 1.287231 | 0.020952 | |
| Cx3cr1 | 3.079479 | 0.020996 | |
| Cux2 | -6.65999 | 0.021046 | |
| Ear6 | -2.15921 | 0.021178 | |
| Nol4l | -1.56723 | 0.021245 | |
| Stag3 | -1.87303 | 0.021329 | |
| Xiap | -0.84854 | 0.022035 | |
| Gm9920 | 4.721133 | 0.022078 | |
| Hgf | -1.85448 | 0.022129 | |
| Chst13 | 4.886891 | 0.022263 | |
| Qpct | 6.061554 | 0.022657 | |
| Gan | -4.84872 | 0.022836 | |
| Armc3 | -1.50885 | 0.022842 | |
| Tmc3 | -2.92336 | 0.023162 | |
| Aff1 | -0.86689 | 0.023443 | |
| Zfp677 | -1.72308 | 0.02351 | |
| Klrb1c | 1.121024 | 0.02392 | |
| Slc16a10 | 1.008564 | 0.023946 | |
| E2f7 | 0.975752 | 0.023954 | |
| Zfp820 | -1.79584 | 0.024092 | |
| Rps6ka6 | 4.998291 | 0.024384 | |
| Bcl2l14 | -2.19586 | 0.024674 | |
| Galnt3 | 5.069969 | 0.025063 | |
| Fam222a | 5.70426 | 0.025265 | |
| Dmxl2 | -2.48532 | 0.025292 | |
| Mt1 | 1.399425 | 0.025856 | |
| Cstad | 3.434279 | 0.026384 | |
| Fam184a | -1.93871 | 0.026469 | |
| Armcx1 | -0.95507 | 0.026473 | |
| Cldn13 | 4.921726 | 0.026777 | |
| Poln | 3.662792 | 0.026801 | |
| Aktip | -0.92886 | 0.026899 | |
| Zan | -2.52406 | 0.027092 | |
| Sytl3 | 5.917821 | 0.027253 | |
| Txlnb | -3.11388 | 0.027301 | |
| Ccdc148 | 5.507459 | 0.027316 | |
| Chga | 3.032545 | 0.027379 | |
| Hivep3 | -0.95032 | 0.027398 | |
| Asns | -1.11864 | 0.02781 | |
| Mfsd9 | -4.21592 | 0.028924 | |
| Ticrr | -1.08152 | 0.029049 | |
| Fam167a | -1.27472 | 0.02923 | |
| Mamdc2 | -0.96127 | 0.029608 | |
| Pabpc1l | -6.6951 | 0.029618 | |
| Ercc6l2 | -1.01362 | 0.029797 | |
| 1190002N15Rik | 1.482483 | 0.030254 | |
| Asb7 | -1.13263 | 0.030619 | |
| Olfm1 | 3.770959 | 0.03096 | |
| Cdh1 | 3.806321 | 0.031003 | |
| Zfp568 | -1.1913 | 0.03122 | |
| Prdm9 | -2.03176 | 0.031607 | |
| Olr1 | 5.75644 | 0.031668 | |
| Trim2 | -4.01633 | 0.032002 | |
| Prom1 | -2.6037 | 0.032366 | |
| Shf | -2.70683 | 0.032469 | |
| Cadm3 | 6.467112 | 0.03268 | |
| Mcpt8 | 1.810416 | 0.032921 | |
| Xpa | -1.62251 | 0.033176 | |
| AI854703 | -1.5777 | 0.033935 | |
| Itgb3 | 1.453625 | 0.034118 | |
| Perp | 2.435804 | 0.034136 | |
| Tmtc3 | -1.14249 | 0.034195 | |
| B3gnt7 | -2.02275 | 0.034342 | |
| Taf4b | 1.371576 | 0.034463 | |
| Tlr12 | 1.651134 | 0.035726 | |
| Nos1ap | 1.614797 | 0.036456 | |
| Nek2 | 1.580203 | 0.036761 | |
| Tti1 | -1.04977 | 0.036826 | |
| Fam208a | -0.96505 | 0.037513 | |
| Bbs10 | 3.468341 | 0.037828 | |
| Tubb6 | 0.905211 | 0.038003 | |
| Zfp438 | -1.78274 | 0.038346 | |
| Hdac8 | -1.06587 | 0.03856 | |
| Nedd1 | -1.29684 | 0.038647 | |
| Cyp39a1 | -1.98663 | 0.03874 | |
| Mdk | -4.11413 | 0.038746 | |
| Cped1 | -3.43636 | 0.039111 | |
| Sulf2 | 1.689236 | 0.039495 | |
| Ear2 | -2.03292 | 0.039637 | |
| Samd9l | -1.00978 | 0.04021 | |
| Tnfrsf19 | -3.37096 | 0.040357 | |
| Cep85l | -2.42467 | 0.040369 | |
| Ascc3 | 0.96905 | 0.040722 | |
| Cd86 | -1.07002 | 0.041256 | |
| Gadd45b | 0.862697 | 0.041343 | |
| Asb13 | -1.01048 | 0.041623 | |
| Chdh | 1.032257 | 0.041661 | |
| Evc2 | -4.31532 | 0.041806 | |
| Prr15 | -2.56776 | 0.04227 | |
| Lrp4 | -1.59607 | 0.042313 | |
| 4930447C04Rik | -1.51031 | 0.042507 | |
| Msl3l2 | -1.68125 | 0.042527 | |
| Ypel4 | -3.85746 | 0.043023 | |
| 1700001O22Rik | -3.41493 | 0.04343 | |
| Cd300lb | 1.424958 | 0.043475 | |
| Axl | -1.4189 | 0.043522 | |
| Kpna2 | 1.257247 | 0.043565 | |
| Map3k9 | -3.20584 | 0.043786 | |
| Slc8b1 | -0.89492 | 0.044049 | |
| C77080 | -0.98808 | 0.044154 | |
| Ccrl2 | -0.97448 | 0.044189 | |
| Mmd | 0.859834 | 0.044273 | |
| Ptgr2 | -0.98904 | 0.044275 | |
| Gcsam | 2.858252 | 0.045099 | |
| Zc3h12a | -1.1777 | 0.045638 | |
| Ms4a4c | 1.16504 | 0.045833 | |
| Agl | -0.98673 | 0.046587 | |
| Slc19a2 | -1.76459 | 0.04661 | |
| Prmt6 | 0.870446 | 0.04666 | |
| Cry1 | 0.986478 | 0.046921 | |
| Cpa3 | 1.129253 | 0.047182 | |
| Cd247 | 1.548259 | 0.04779 | |
| Serpine2 | 4.739998 | 0.048063 | |
| Slc35f5 | -1.14805 | 0.048695 | |
| Klrd1 | 3.108488 | 0.048806 | |
| Smim13 | 1.556259 | 0.048829 | |
| Il1r2 | 1.29681 | 0.049002 | |
| Ppm1l | 1.306415 | 0.049363 | |
| Slc10a7 | 0.986387 | 0.049565 | |
| Sgip1 | -2.63958 | 0.049756 | |
| Ctnnd2 | 2.431942 | 0.049973 | |
